# Seizure Induced by Defecation in a 15-Year Old Autistic Patient: A Case Report and Literature Review

**Published:** 2020

**Authors:** Mohammad Hasan MOHAMMADI, Iraj SHAHRAMIAN, Ali BAZI, Mojtaba DELARAMNASAB

**Affiliations:** 1 Professor of Pediatric Neurology, Zabol University of Medical Sciences, Zabol, Iran; 2 Pediatric Gastroenterology and Hepatology Research Center, Zabol University of Medical Sciences, Zabol, Iran; 3 Hematology, Faculty of, Zabol University of Medical Sciences, Zabol, Iran,; 4 Nursing Faculty of, Zabol University of Medical Sciences, Zabol, Iran,

**Keywords:** Reflex seizure, Defecation, Autism, Electroencephalography

## Abstract

Epilepsy in autism is a relatively common phenomenon. However, reflex seizures provoked by multifactorial stimuli are rare in these patients. We here reported the first case of defecation-induced seizure in a 15-year old autistic girl. The patient had been diagnosed with epilepsy within the first year after birth; however, seizures induced by bowel movements were observed at the age of 15. Reflex seizures showed a myoclonic pattern represented with one-sided neck deflection. EEG showed an abnormal polyspike and wave pattern during defecation while the patterns were normal between the attacks. The patient was partially responsive to adrenocorticotropic hormone therapy with a reduced frequency of both reflexes and generalized seizures. Phenobarbital therapy was effective to manage recurrent seizure attacks. Although seizure is commonly encountered in autism, reflex seizures induced by defecation have not been previously reported in this condition.

## Introduction

Epilepsy is the most encountered neurological disorder affecting 0.5-1% of the world’s population (1). In the recent refined guidelines published by the International League Against Epilepsy (ILAE), more attention has been dedicated to reflex seizures in the definition of epilepsy (2). According to the new definition, either a single reflex seizure with the risk of recurrence or two reflex seizures separated occurring more than 24 h apart can be considered as an epileptic syndrome (2, 3).

Reflex seizures may be induced by various stimuli, such as eating, listening to music, playing computer games, swimming in hot water, exposure to the light, as well as shock, orgasm, and a variety of intellectual activities (4-6). Stress, visual stimuli, sleep disorders, and fatigue have been reported as the most frequent factors to induce reflex seizures (6-8). Although neurological and gastrointestinal systems are interrelated (9), reflex seizures triggered by digestive stimuli are relatively infrequent. In particular, reflex seizure following defecation represents an extremely rare phenomenon with only two reports of reflex seizures linked to defecation (10, 11).

Autism is a multifactorial mental condition characterized by abnormalities in social communications (12). Epilepsy is associated with a relatively common adverse outcome in autism. The concurrent epileptic phenotype in autistic patients may further weaken communication skills and reduce life expectancy (13). Although seizures are common in autistic patients with a frequency of 5-38% (12, 14), reflex seizures are rare in autism. Here, we reported the first case of reflex seizure provoked by defecation in a teenager with autism.

## Case Report

A 15-year old girl, weighing 24 kg, admitted to our hospital because of refractory reflex seizure induced by defecation. The patient had been previously diagnosed with autism within the first year of life. The patient had developmental tardiness with independent walking ability at five years of age. The patient had also problems with writing and speaking skills. The speech skill was severely flawed as only 3-4 words could be spoken at the time of patient admission. She had a history of generalized seizures when she was one year old. 

In clinical examination, no pulmonary or cardiovascular dysfunctions were reported. Also, the functional assessment of the neurological system revealed normal findings. Vital signs were within the normal range (blood pressure; 85/50 mmHg, pulse; 120 BPM, and body temperature; 36.5 ℃). There was no splenomegaly or hepatomegaly. The patient was conscious during the initial examination and remained conscious through the course of the disease. Rectal distension and micturition and defecation were recorded at admission. Upper and lower limb deformities were observed. During the initial evaluation, the patient’s parents stated weight loss over the past three months accompanied by the loss of appetite. There was no history of drug or food sensitivities or other allergic reactions. 

At the time of admission, cell blood counts were within normal range except for a low level of hemoglobin (11.5 g/dl) and hematocrit (35.9%). Fasting blood glucose was 62 mg/dl, erythrocyte sedimentation rate (ESR) was normal, and C-reactive protein (CRP) was negative. 

Reflex seizures had been developing during bowel movements. Defecation-induced reflex seizures were associated with a myoclonic pattern presenting with nonrhythmic extension and flexion movements. The duration of reflex seizure attacks was variable (10-60 s). The attacks were reported as at least 10 episodes per day at the time of admission. 

**Therapeutic interventions**

The patient had been under treatment with different monotherapy and combinational therapy regimens using variable drugs, including Topiramate, Clobazam, Lamotrigine, L- carnitine, Nitrazepam, Depakene (sodium valproate), *Clonidine, and Pantoprazole. As monotherapy and combinational therapy with two drugs did not affect the disease presentations, the patient had been administrated with a tetra-drug regimen (sodium valproate + *Lamotrigine + Clobazam + Topiramate) continued until the time of admission. 

**Figure 1:**
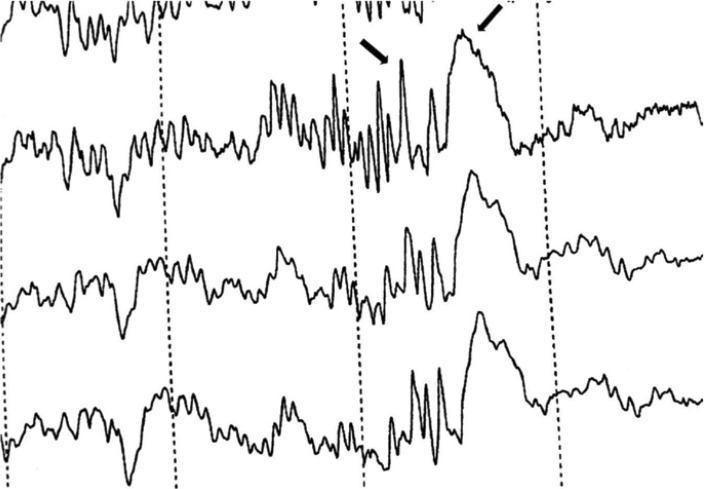
The electroencephalography showing a polyspike and wave pattern during a defecation-induced seizure in an autistic girl

After admission, to reduce the pressure during defecation, we administrated Lactolose (1 ml/kg every 8 h). However, the seizures continued to occur even with loose and smooth defecations. The therapeutic intervention included intramuscular adrenocorticotropic hormone (ACTH) at the dose of 50 U at the time of admission. The patient was carefully monitored during the first two hours following ACTH administration. ACTH therapy was discontinued after two days due to high blood pressure and restored with the initial dose after normalization of blood pressure on day four. The patient also received methylprednisolone pulse therapy (30 mg/kg) for 5 days. Electroencephalography (EEG) was performed five days after admission and during defecation revealing a polyspike and wave pattern (Figure 1). 

Despite using a combination of the above-mentioned anti-epileptic therapeutics, the patients continued to develop reflex seizures. Ten days after admission, phenobarbital was initially infused at the dose of 200 mg and continued at the dose of 100 mg daily. The patient experienced improvements in the frequency of seizure attacks following phenobarbital therapy. The patient was finally discharged prescribing the pre-admission therapeutic regimen 11 days after admission.

## Discussion

This was the first report of a 15-year old autistic girl presenting with defecation-induced myoclonic reflex seizures. The defecation-induced reflex seizure is an extremely rare phenomenon previously reported in only two cases (10, 11). The first case was a 5-year-old girl (11), and the second was a 9-year-old boy (10). Similar to our patient, reflex seizures were followed by juvenile myoclonic epilepsy in both cases (10, 11). At the intervals between seizure attacks, no EEG abnormality was detected; however, an abnormal polyspike wave in the left frontotemporal region was observed in the EEG pattern during seizures. This was also similar to the EEG pattern observed in the previously reported cases (10, 11). 

In a previous case of seizure provoked by defecation, the attacks occasionally occurred within 1-2 min following bowel movement but not during the activity (10)., which is not consistent with our case, as the seizures were initiated during defecation. In their reports, Harbord et al. described a case experiencing generalized seizure attacks intermittently (every 3-4 days) associated with reflex seizures (10). Both reflex and generalized seizure episodes subsided in response to carbamazepine (11) and lamotrigine (10) in the previously reported cases of defecation-induced seizure. In the present case, ACTH therapy relieved the seizure attacks. After seizure recurrence ten days after hospitalization, phenobarbital was effective to reduce the attacks. However, the seizure attacks were refractory to common anti-epileptic treatments. A partial response to common anti-epileptic drugs in our case was probably due to the autism in our case, which is known to be associated with drug-refractory seizures (15). 

It seems that the intestinal movements can stimulate the temporal region of the brain, which subsequently leads to seizure attacks. It has been also reported that the supplementary motor area (SAM) receives neurological signals provoked by sphincter activity during a bowel movement (16). In parallel, the contraction of the particular muscles, including sphincter in the pelvis has been noted to activate the SAM region (17). These suggest that the contraction of such muscles may be the potential stimulators of reflex seizures during defecation. More studies are needed to explore the neurological pathways connecting defecation to seizure and epilepsy.

Autism is commonly associated with epilepsy (14). Autism and epilepsy share two common pathological features; 1- diminished inhibitory cycles of minicolumn, and 2- reduced inhibitory effects of gamma-aminobutyric acid (GABA). These effects are known to predispose the brain to stimulatory factors, which subsequently leads to seizures due to the higher ratio of stimulatory-to inhibitory signals within the cortex (14). Besides, the development of epilepsy and seizure in autism may also be related to nutritional factors (18). Furthermore, a role has been proposed for inflammation as a triggering factor of epilepsy in autism (19). In our patient, however, inflammatory markers, including CRP and ESR were within normal ranges at admission and did not change in the course of the disease. The potential contributors intercalating autism and seizure are yet to be identified. 

**In Conclusion, **Reflex seizure induced by defecation is a rare clinical condition. In autistic patients, defecation-provoked reflex seizures can present with partial refractory epilepsy requiring multiple therapeutic strategies. Regarding a high prevalence of various types of seizures in autism, it is recommended to carefully monitor these patients for identifying possible triggering factors for the seizure attacks.
